# Quantifying reef-derived sediment generation: Introducing the SedBudget methodology to support tropical coastline and island vulnerability studies – Addendum

**DOI:** 10.1017/cft.2023.19

**Published:** 2023-07-04

**Authors:** Chris T. Perry, Ines D. Lange, Marleen Stuhr

**Affiliations:** 1 Geography, Faculty of Environment, Science and Economy, University of Exeter, Exeter, UK; 2 Biogeochemistry and Geology, Leibniz Centre for Tropical Marine Research, Bremen, Germany

The data entry sheets that can be used with the SedBudget methodology can be found at: https://geography.exeter.ac.uk/sedbudget/. Copies of these with the field data collected in this study are available from the corresponding author, Chris T. Perry.
